# The efficacy and safety of Tisotumab vedotin in the treatment of recurrent/metastatic cervical cancer: a systematic review and meta-analysis of single-arm studies

**DOI:** 10.3389/fphar.2025.1538980

**Published:** 2025-05-19

**Authors:** Siyuan Zeng, Xin Li, Simin Xiao, Peina Yang, Changsheng Lin, Huiling Chen, Hu Zhao, Jun Zhan, Xue Xiao

**Affiliations:** ^1^ Department of Gynecology and Obstetrics, West China Second University Hospital, Sichuan University, Chengdu, China; ^2^ Key Laboratory of Birth Defects and Related Diseases of Women and Children, Ministry of Education, West China Second Hospital, Sichuan University, Chengdu, China; ^3^ Department of Medical Genetics and Prenatal Diagnostic Center, West China Second University Hospital, Sichuan University, Chengdu, China; ^4^ Department of Radiology, Xin Du Hospital of Traditional Chinese Medicine, Chengdu, China

**Keywords:** Tisotumab vedotin, recurrent, metastatic, cervical cancer, antibodydrug conjugate, prognosis

## Abstract

**Objective:**

This study was conducted to evaluate the efficacy and safety of Tisotumab Vedotin (TV) in the treatment of recurrent/metastatic cervical cancer (r/m CC) through a systematic review and meta-analysis.

**Methods:**

The clinical studies on the monotherapy of TV for r/m CC were retrieved comprehensively from some databases, including PubMed, Embase, and Cochrane Library. The inclusion criteria encompassed observational studies and randomized controlled trials. Data analysis was performed using STATA 15.0. Besides, the median overall survival (OS), median progression-free survival (PFS), disease control rate (DCR), objective response rate (ORR), and the incidence of adverse events (AEs) and AEs at Grades 3–5 were calculated.

**Results:**

A total of 5 articles (covering 7 studies and 527 patients) were included in this study. The meta-analysis results revealed that the median OS, median PFS, ORR, and DCR were 11.83 months, 4.22 months, 29.9%, and 75.1%, respectively, for patients treated with TV. The incidence of AEs was 99.1%, and AEs at Grades 3–5 were reported in 61.7% of patients.

**Conclusion:**

TV demonstrates significant efficacy as a second-line or third-line therapy for r/m CC, making it a promising therapeutic option. Nevertheless, large-scale randomized controlled trials are needed to validate these findings and optimize clinical application strategies.

**Systematic Review Registration:**

https://www.crd.york.ac.uk/PROSPERO/view/CRD42024577046, identifier CRD42024577046.

## 1 Introduction

Cervical cancer (CC) is a malignant tumor that poses a serious threat to female health. Although CC can be effectively treated through surgery and radiotherapy, a subset of patients may still experience recurrence or metastasis (Sung et al., 2021). These patients with recurrent or metastatic cervical cancer (r/m CC) are prone to a poor prognosis, and treatment options are very limited. It has been indicated that approximately one-third of patients with r/m CC exhibit a short-term response to platinum-based chemotherapy; however, the median survival for most patients is only 7–12 months (Liontos et al., 2019; Wright et al., 2019).

Although such immune checkpoint inhibitors as programmed death-1 (PD-1) inhibitors combined with chemotherapy have become the first-line therapy for programmed death-ligand 1 (PD-L1) positive patients with r/mCC, there is still a lack of standardized regimens for second- and third-line therapies, accompanied by a low response rate (objective response rate [ORR] < 15%) (Monk et al., 2022). On 20 September 2021, the U.S. Food and Drug Administration (FDA) approved Tisotumab Vedotin (TV) for the treatment of r/m CC in later-line therapies (Bogani et al., 2023).

Antibody-drug conjugates (ADCs) can combine monoclonal antibodies with cytotoxic drugs to achieve targeted cancer cell destruction (Dumontet et al., 2023). TV is an investigational ADC that targets tissue factors, which are highly expressed in various solid tumors, including CC. As one of the ADC-based drugs, TV has garnered substantial data supporting its use in the treatment of CC, demonstrating superior efficacy in second-line therapy compared with conventional chemotherapy (Song et al., 2022; Markham, 2021). According to the latest National Comprehensive Cancer Network (NCCN) guidelines published in 2024, TV has been elevated from an alternative second-line therapy to a preferred option (Abu-Rustum et al., 2023).

Although there have been a few studies on the use of TV in the treatment of r/m CC, the reliability of clinical data has been challenged due to the small sample size and inconsistent conclusions (Hong et al., 2020; Yonemori et al., 2022; Vergote et al., 2023; Vergote et al., 2024; Coleman et al., 2021). Therefore, there is an urgent demand for a systematic review and meta-analysis to evaluate the efficacy and safety of TV in the treatment of r/m CC, thus providing more reliable evidence for clinical practice.

## 2 Methods

### 2.1 Study design and registration

This study was conducted following the principles of the Preferred Reporting Items for Systematic Reviews and Meta-Analyses (PRISMA) and has been registered in the International Prospective Register of Systematic Reviews, with the registration number being CRD42024577046 (Booth et al., 2012; Page et al., 2021).

### 2.2 Inclusion criteria

#### 2.2.1 Study types

Observational studies and randomized controlled trials (RCTs).

#### 2.2.2 Participants

Patients with r/m CC, regardless of race, nationality, or pathological findings, who did not receive any other antitumor drugs during treatment.

#### 2.2.3 Intervention

Monotherapy with TV.

#### 2.2.4 Outcome measures

Efficacy Outcomes included the median overall survival (OS), median progression-free survival (PFS), disease control rate (DCR), and objective response rate (ORR). Safety outcomes included the incidence of AEs and AEs at Grades 3–5 during treatment.

### 2.3 Exclusion criteria

Duplicate publications, reviews, study protocols, case reports, or studies without relevant outcome measures and relevant data.

### 2.4 Literature retrieval strategy

Relevant studies were retrieved from several databases, including PubMed, Embase, and Cochrane Library from their establishment to August 2024. The retrieval aimed to identify clinical trials on the use of TV monotherapy in patients with r/mCC. The retrieval was performed in combination with subject headings and free text terms, and the key English retrieval terms included Tisotumab Vedotin, Antibody-Drug Conjugate, Cervical Cancer, Recurrent, Metastatic, Uterine Cervical Neoplasms, Objective Response Rate, and Adverse Events.

### 2.5 Literature screening, data extraction, and quality assessment

Two reviewers independently collected and screened the literature according to the inclusion and exclusion criteria, extracted data, and cross-checked the information. A third party was consulted to resolve any disagreement through discussion. During literature screening, abstracts were first reviewed to exclude obviously irrelevant studies, followed by a full-text review to determine final studies. The extracted data included basic study information (study title, first author, publication date, etc.) and characteristics of the study population (number of cases, median age, outcome measures, treatment regimen, median follow-up time, etc.). The quality of the studies was assessed using the Methodological Index for Non-Randomized Studies (MINORS) scale (Zeng et al., 2015). To further explore potential sources of heterogeneity and assess the robustness of our findings, subgroup analyses were conducted across multiple clinical outcomes, including ORR, DCR, PFS, and AEs at Grades ≥3. Besides, the included studies were stratified based on key design and methodological characteristics, including study type (multicenter vs. single-center), sample size (>50 vs. <50), and methodological quality (MINORS score ≥15 vs. 14). Within each subgroup, pooled estimates were calculated, and heterogeneity was assessed using the I^2^ statistic along with the corresponding P values for interaction.

### 2.6 Statistical analysis

Meta-analysis was performed using Stata 15.0, and the effect sizes of the median OS, median PFS, ORR, DCR, and the incidence of AEs and AEs at Grades 3–5 were calculated. Heterogeneity was assessed using the χ^2^ test, with significance levels set at P = 0.1 and I^2^ = 50%. A fixed-effects model was used when P > 0.1 and I^2^ ≤ 50%; a random-effects model was used when P ≤ 0.1 and I^2^ > 50%. Statistical significance was defined as P < 0.05.

## 3 Results

### 3.1 Literature screening process and results

**FIGURE 1:**
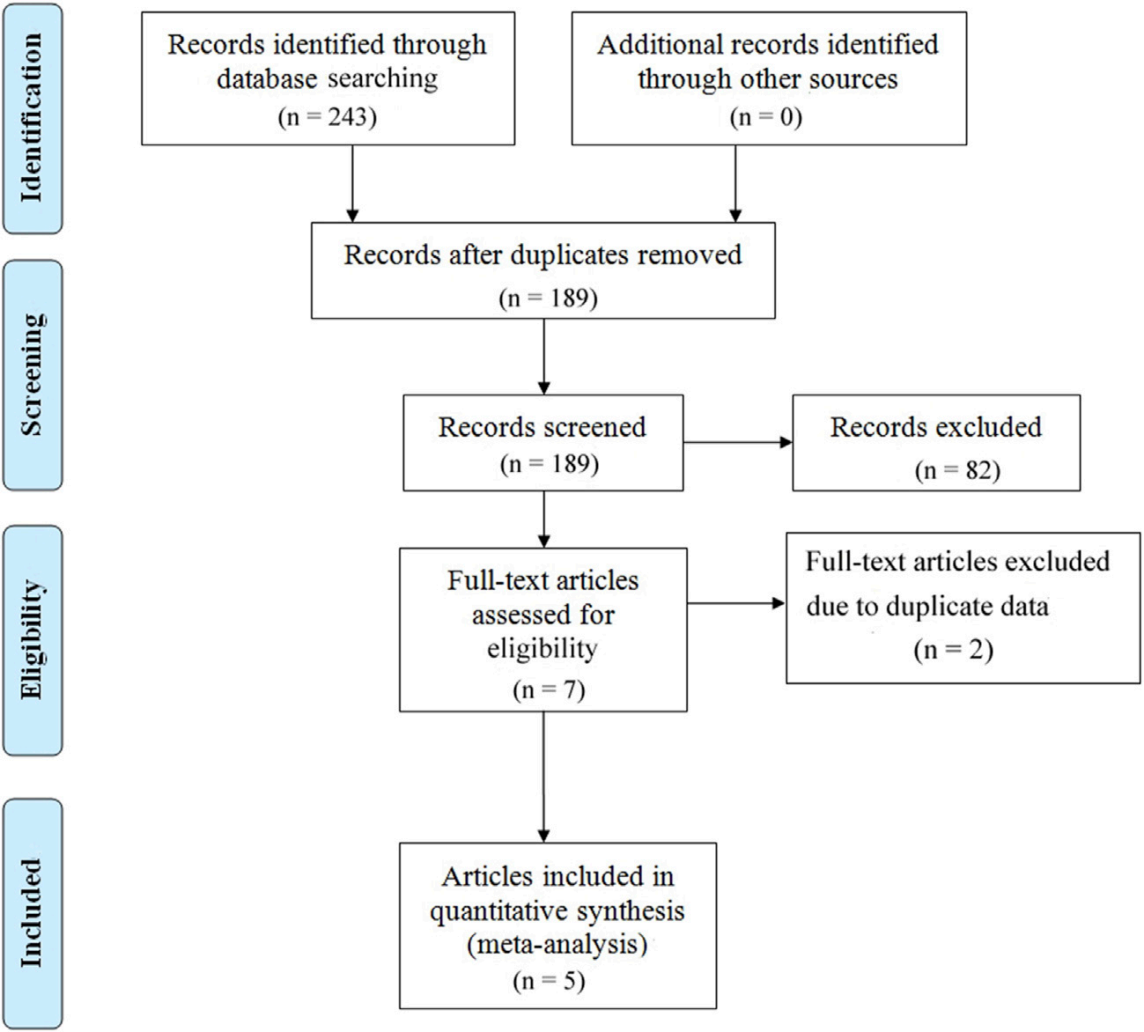
Flow plot of the literature selection process.

A total of 243 articles were initially screened. After removing duplicates, 189 articles were retained. After further screening and exclusion of studies that did not meet the inclusion criteria, 5 articles (covering 7 studies) were ultimately included in the analysis (Figure 1).

### 3.2 Basic characteristics of included studies

Five articles (covering 7 studies) were included, of which 4 were single-arm clinical studies and 1 was a randomized controlled trial based on the efficacy comparison between the monotherapy of TV or the combined therapy of TV and other chemotherapy drugs. All studies were analyzed using single-group rates. The sample sizes ranged from 17 to 253 patients, with a total of 527 patients involved. The average age of the patients ranged from 46 to 51 years. In all studies, the dosing regimen was 2.0 mg/kg every 3 weeks (Table 1). The MINORS scoring system indicated that the studies Hong 2019, Coleman 2021, and Yonemori 2022 each scored 15 points, reflecting high methodological quality, despite lacking scores related to control groups. Vergote 2023 scored 14 points, slightly lower than that of previous studies. In contrast, Vergote 2024 achieved the highest score of 23 points, indicating superior methodological quality with high scores across all relevant criteria (Table 2).

**TABLE 1:** Characteristics of studies included in this meta-analysis.

Contry	Identifier	Sample size	Median age	Median follow-up (months)	Interventions	Median OS with 95% CI (months)	Median PFS with 95% CI (months)
United States	NCT02001623	55	46 (21–73)	3.5 (0.6–11.8)	2.0 mg/kg every 3 weeks	NR	4.1 (1.7–6.7)
Europe and the United States	NCT03438396	101	50 (43–58)	10	2.0 mg/kg every 3 weeks	12.1 (9.6–13.9)	4.2 (3.0–4.4)
Japan	NCT03913741	17	47 (33–66)	6.3 (1–12)	2.0 mg/kg every 3 weeks	11.4 (6.2-not reached)	3.1 (1.2–7.1)
Multicenter	NCT03786081	33	51 (25–78)	17.8 (1–26)	2.0 mg/kg every 3 weeks	NR	6.9 (4.0–11.1)
Multicenter	NCT03786081	33	47 (29–76)	21.7 (1–29)	2.0 mg/kg every 3 weeks	NR	5.3 (4.0–12.2)
Multicenter	NCT03786081	35	47 (31–73)	15.0 (1–29)	2.0 mg/kg every 3 weeks	15.3 (9.9 to NR)	5.6 (2.7–14.2)
Multicenter	NCT04697628	253	51 (26–80)	10.8	2.0 mg/kg every 3 weeks	11.5 (9.8–14.9)	4.2 (4.0–4.4)

**TABLE 2:** Quality assessment of included studies using the MINORS scoring system.

Studies	A clearly stated aim	Inclusion of consecutive patients	Prospective collection of data	Endpoints appropriate to the aim of the study	Unbiased assessment of the study endpoint	Follow-up period appropriate to the aim of the study	Loss to follow up less than 5%	Prospective calculation of the study size	An adequate control group	Contemporary groups	Baseline equivalence of groups	Adequate statistical analyses	Scores
Hong 2019	2	1	2	2	2	2	2	2	NA	NA	NA	NA	15
Coleman 2021	2	1	2	2	2	2	2	2	NA	NA	NA	NA	15
Yonemori 2022	2	1	2	2	2	2	2	2	NA	NA	NA	NA	15
Vergote 2023 1	2	1	2	2	1	2	2	2	NA	NA	NA	NA	14
Vergote 2023 2	2	1	2	2	1	2	2	2	NA	NA	NA	NA	14
Vergote 2023 3	2	1	2	2	1	2	2	2	NA	NA	NA	NA	14
Vergote 2024	2	1	2	2	2	2	2	2	2	2	2	2	23

### 3.3 Meta-analysis results

#### 3.3.1 Efficacy outcomes

##### 3.3.1.1 Median OS

**FIGURE 2:**
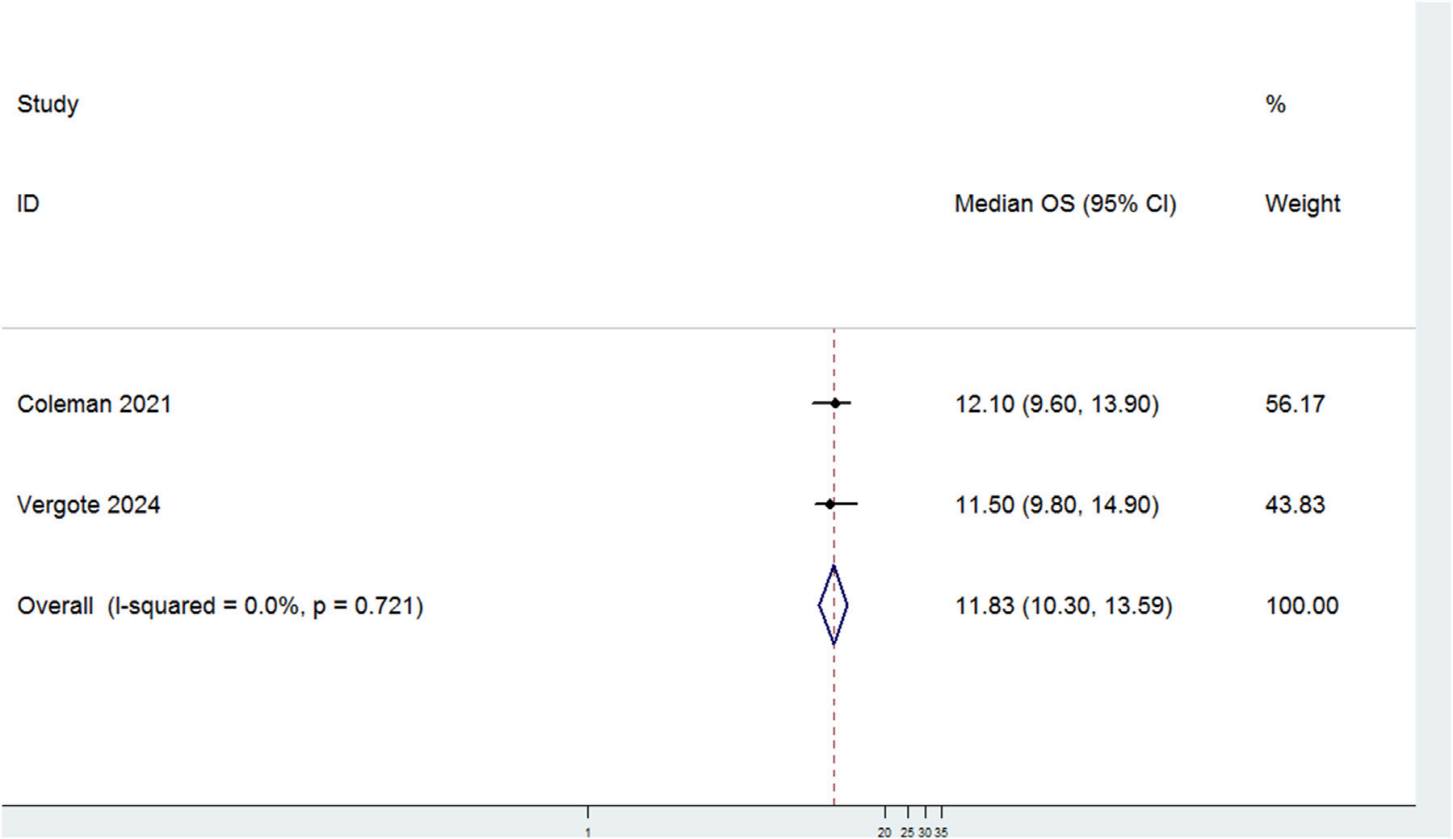
Forest plot of the median overall survival (OS) in patients treated with Tisotumab Vedotin (TV).

Two studies evaluated the median OS of patients with r/mCC treated with TV (Figure 2). There was no significant heterogeneity among the studies (P = 0.721, I^2^ = 0.0%). Therefore, a fixed-effects model was used for the meta-analysis. The results showed that the median OS for patients treated with TV was 11.83 months (95% CI: 10.30–13.59).

##### 3.3.1.2 Median PFS

**FIGURE 3:**
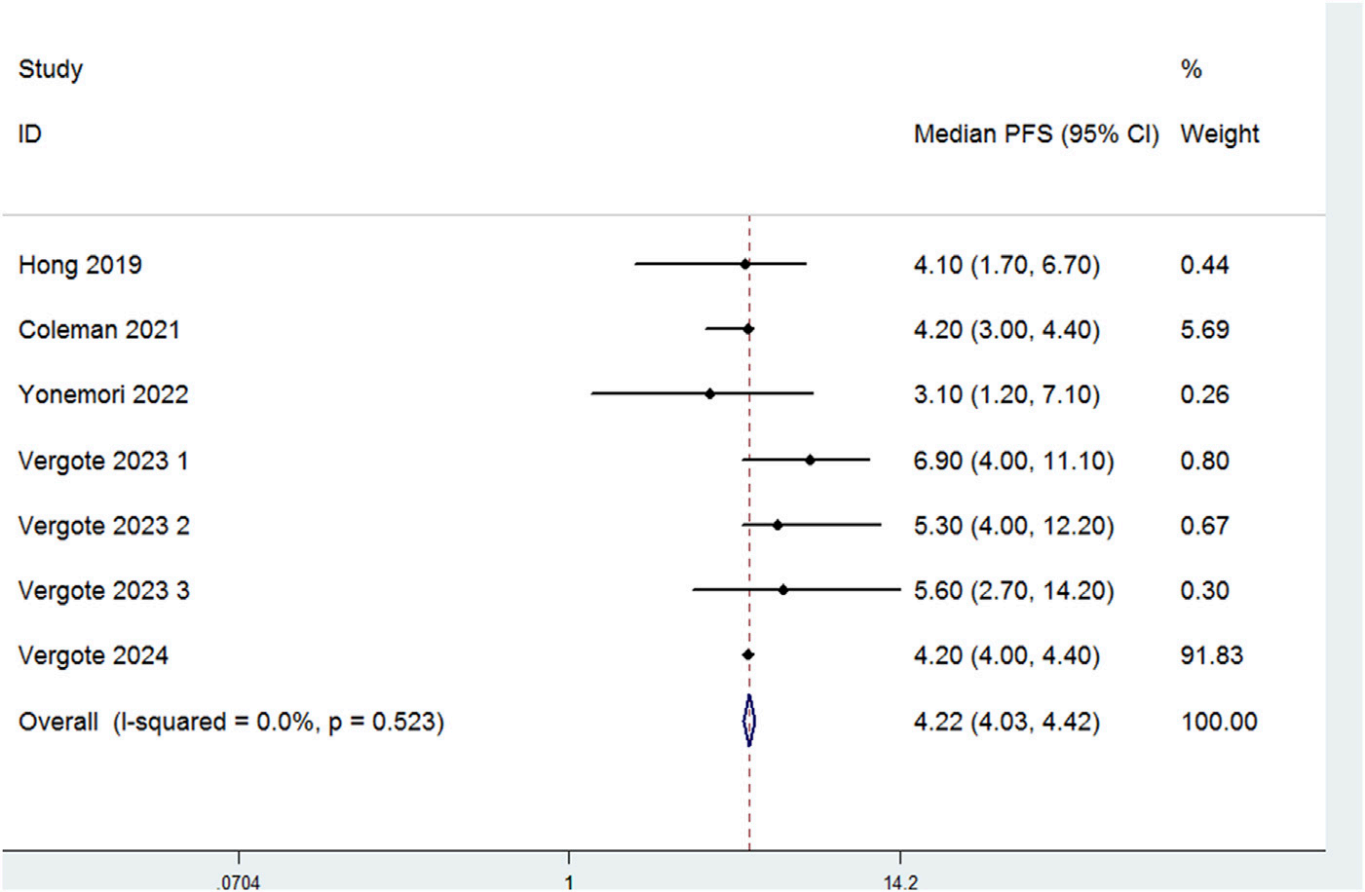
Forest plot of the median progression-free survival (PFS) in patients treated with TV.

Seven studies evaluated the median PFS of patients treated with TV (Figure 3). There was no significant heterogeneity among the studies (P = 0.523, I^2^ = 0.0%). Therefore, a fixed-effects model was used for the meta-analysis. The results indicated that the median PFS for patients treated with TV was 4.22 months (95% CI:4.03–4.22).

##### 3.3.1.3 ORR

**FIGURE 4:**
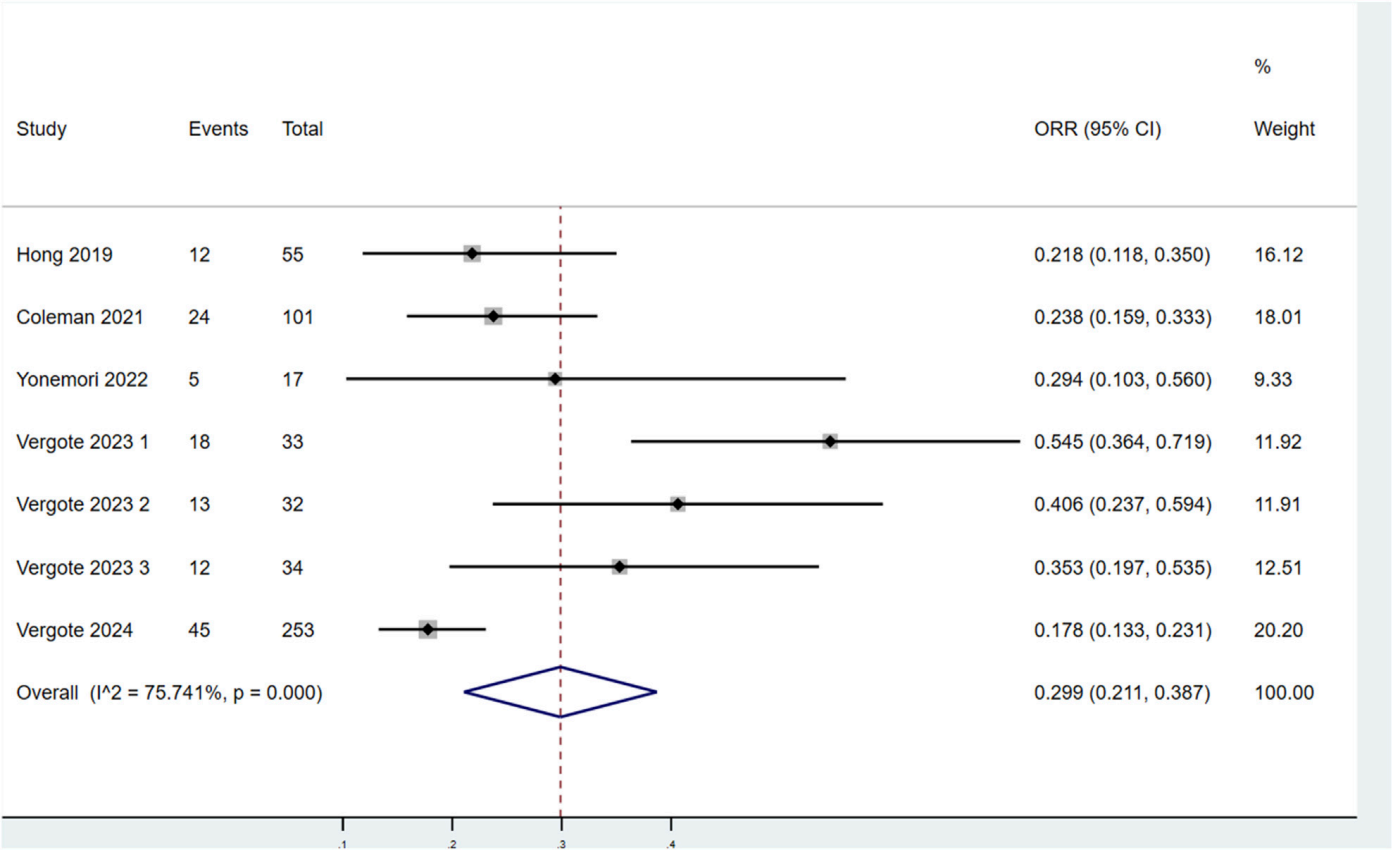
Forest plot of the objective response rate (ORR) in patients treated with TV.

Seven studies assessed the ORR of patients treated with TV (Figure 4). Significant heterogeneity was observed among the studies (P < 0.1, I^2^ = 75.741%). Therefore, a random-effects model was selected for the meta-analysis. The results demonstrated that the ORR for patients treated with TV was 29.9% (95% CI: 21.1%–38.7%).

##### 3.3.1.4 DCR

**FIGURE 5:**
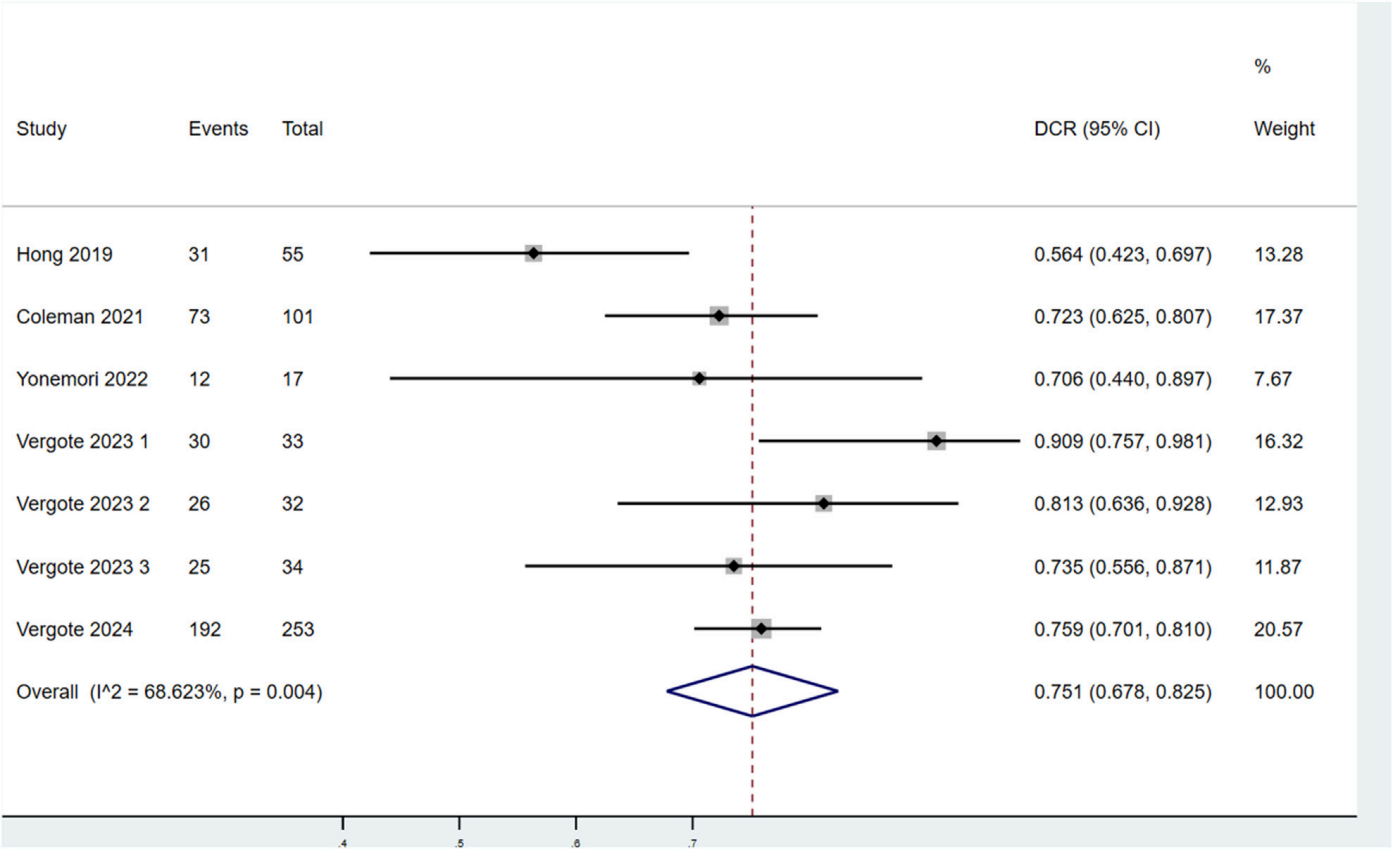
Forest plot of the disease control rate (DCR) in patients treated with TV.

Seven studies evaluated the DCR of patients treated with TV (Figure 5). Significant heterogeneity was observed among the studies (P < 0.1, I^2^ = 68.623%). Therefore, a random-effects model was used for the meta-analysis. The findings showed that the DCR of patients treated with TV was 75.1% (95% CI: 67.8%–82.5%).

#### 3.3.2 Safety outcomes

##### 3.3.2.1 Incidence of AEs

**FIGURE 6:**
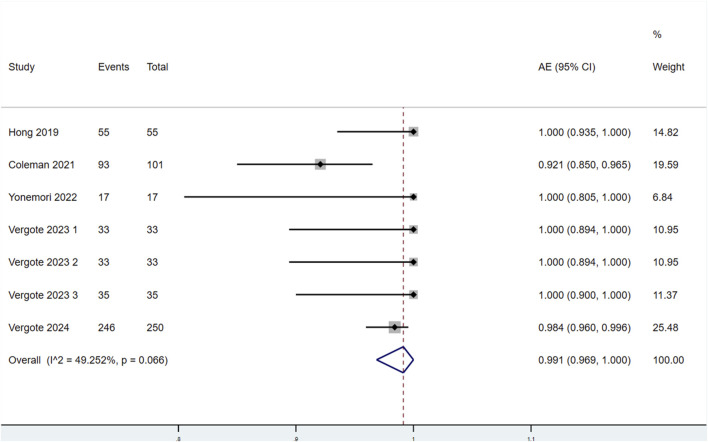
Forest plot of the incidence of adverse events (AEs) in patients treated with TV.

Seven studies reported the incidence of AEs in patients treated with TV (Figure 6). Significant heterogeneity was observed among the studies (P < 0.1, I^2^ = 49.252%). Therefore, a random-effects model was used for the meta-analysis. The results indicated that the incidence of AEs in patients treated with TV was 99.1% (95% CI: 96.9%–100.0%).

##### 3.3.2.2 Incidence of AEs at Grades 3–5

**FIGURE 7:**
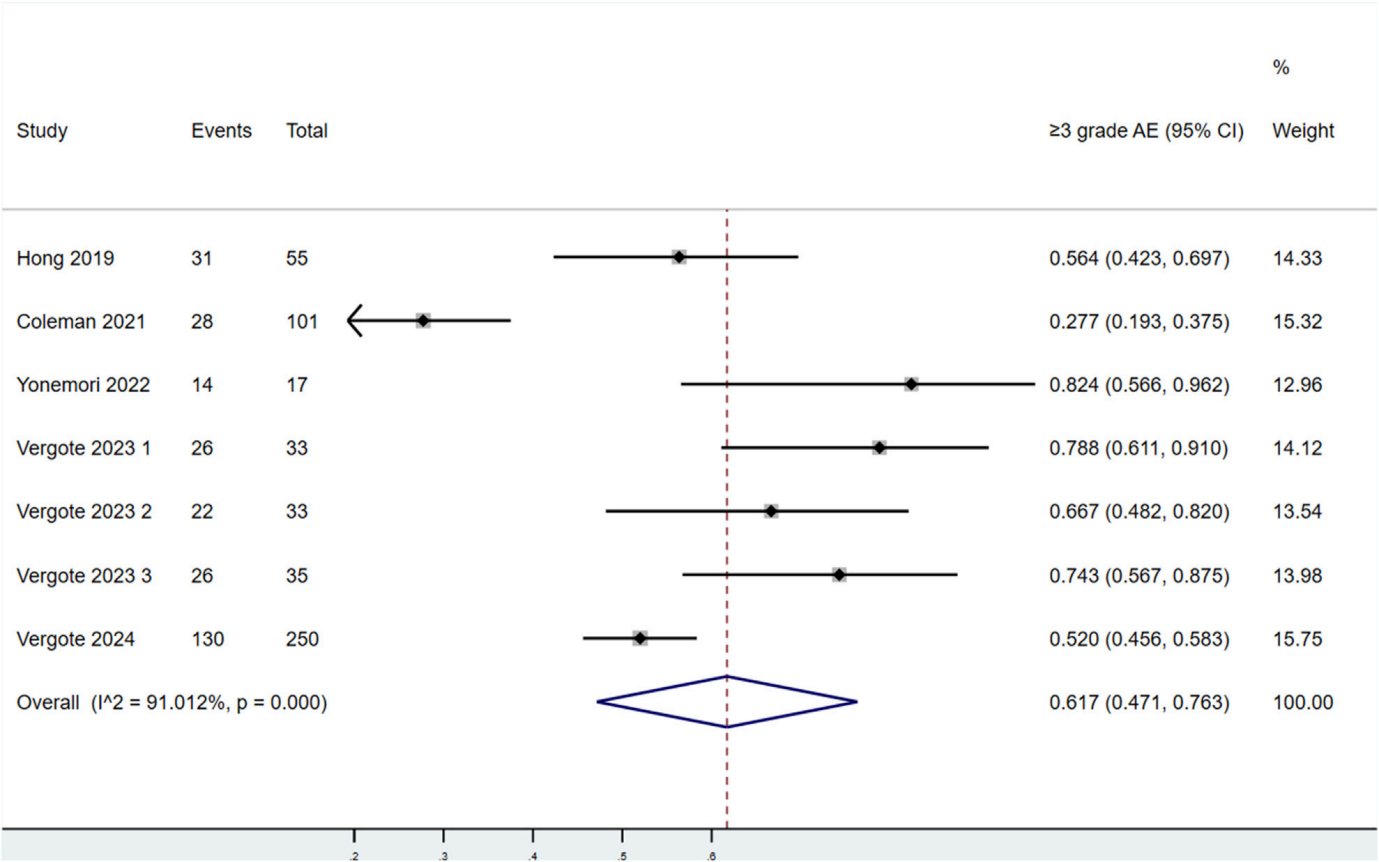
Forest plot of the incidence of adverse events (AEs) at Grades 3–5 in patients treated with TV.

Seven studies evaluated the incidence of AEs at Grades 3–5 in patients treated with TV (Figure 7). Significant heterogeneity was found among the studies (P < 0.1, I^2^ = 91.012%). Therefore, a random-effects model was used for the meta-analysis. The results showed that the incidence of AEs at Grades 3–5 in patients treated with TV was 61.7% (95% CI: 47.1%–76.3%).

##### 3.3.2.3 Subgroup analysis

Subgroup analyses were conducted for ORR, DCR, AEs at Grades ≥3, and PFS to explore the potential sources of heterogeneity. The pooled ORR was 32.4% in multicenter studies and 23.4% in single-center studies. The studies with smaller sample sizes (<50 patients) and those with lower methodological quality (MINORS score = 14) tended to report higher ORR estimates. Similar trends were observed for DCR, with higher rates in studies with smaller sample sizes (81.3%) and those with lower quality scores (83.1%). AEs at Grades ≥3 were consistently high across all subgroups, ranging from 97.7% to 100%, regardless of study design or sample size. The pooled median PFS was 4.23 months in multicenter studies and 3.69 months in single-center studies. Studies with smaller sample sizes and lower quality scores also reported longer median PFS (5.53 and 6.03 months, respectively). No significant subgroup interactions were identified, and heterogeneity remained low to moderate in most comparisons. These findings suggested that study characteristics such as sample size and methodological quality may influence efficacy outcomes, while safety outcomes were uniformly high (Supplementary Table S1).

## 4 Discussion

The results of this study demonstrated that TV was highly effective in the second-line and third-line treatment of r/m CC. The meta-analysis results corroborated that the median OS, median PFS, ORR, and DCR were 11.83 months, 4.22 months, 29.9%, and 75.1%, respectively, in patients treated with TV. In terms of safety, although there was a relatively high incidence of AEs, the incidence of severe AEs was lower in the monotherapy using TV compared with conventional chemotherapy. Hence, TV may be considered a relatively safer treatment option for patients with r/m CC.

R/m CC progresses rapidly with a poor prognosis, making it a leading cause of death in patients with CC. Current chemotherapy, immunotherapy, and targeted therapies cannot satisfy clinical needs (Cohen et al., 2019). In the KEYNOTE-826 study, the regimen of paclitaxel + cisplatin/carboplatin ± bevacizumab combined with pembrolizumab was compared that without pembrolizumab in terms of their efficacy. It was found that pembrolizumab combined with standard treatment significantly improved the ORR in the first-line treatment of patients with CC (65.9% vs. 50.8%) and greatly extended their OS (18.0 months vs. 10.4 months) (Monk et al., 2023). Based on these findings, the NCCN guidelines included paclitaxel + cisplatin/carboplatin ± bevacizumab combined with pembrolizumab (for PD-L1-positive patients) as a recommended first-line treatment for r/m CC. For PD-L1-negative patients, the regimen of paclitaxel + cisplatin/carboplatin ± bevacizumab was still recommended (Abu-Rustum et al., 2023). TV, as an emerging targeted therapy, has been incorporated into the NCCN guidelines for the second-line treatment of recurrent CC (Abu-Rustum et al., 2023). The reported median OS (11.83 months) and PFS (4.22 months) for TV in our meta-analysis were indeed promising and consistent with the findings of the phase 3 innovaTV 301 trial, which demonstrated a median OS of 11.5 months and PFS of 4.2 months in the TV group, significantly outperforming investigator’s choice chemotherapy (median OS 9.5 months; PFS 2.9 months) (Vergote et al., 2024). In contrast, pembrolizumab-based regimens - although approved as first-line options for PD-L1-positive patients - showed more modest efficacy in second-line settings. For instance, in the KEYNOTE-158 study (Marabelle et al., 2020), pembrolizumab monotherapy yielded an ORR of only 14.3% with no significant OS improvement in the general r/mCC population. Additionally, some recent data from the innovaTV 205 trial showed that combining TV with pembrolizumab in second-/third-line therapy achieved an ORR of 35.3%, higher than either agent alone, with the median duration of response being 14.1 months (Vergote et al., 2023). Therefore, current evidence suggests that TV monotherapy provides superior or at least comparable efficacy compared with existing second-/third-line therapies, particularly for patients who have progressed on immunotherapy or are ineligible for this therapy (Vergote et al., 2024). Subgroup analyses from the included studies suggested some variations in efficacy based on patient characteristics, although detailed individual-level data were limited in our meta-analysis. Notably, in the phase 3 innovaTV 301 trial, the survival benefit of TV was generally consistent across key subgroups, including age, geographic region, prior bevacizumab exposure, and prior immunotherapy use (Vergote et al., 2024). For instance, both immunotherapy-naive and pretreated patients obtained comparable OS benefits from TV. However, the expression of PD-L1 is not a stratification factor in most TV trials, and current data do not support a clear predictive role of PD-L1 for TV efficacy. Similarly, the innovaTV 205 study (TV + pembrolizumab) reported antitumor activity, which was irrelevant to the expression of PD-L1 (Vergote et al., 2023).

The acting mechanism of ADCs in tumor treatment differs from that of conventional chemotherapy and immunotherapy and involves several key processes. (1) Specific Targeting and Cytotoxic Drug Release: The monoclonal antibodies in ADCs specifically recognize and bind to corresponding tumor cell antigens, followed by cellular internalization. Once entering the cell, the cytotoxic drug attached to the antibody is released under the action of the specific potential of hydrogen or enzymes, directly targeting cancer cells (Kovtun et al., 2010). (2) Antibody-dependent Cell-mediated Cytotoxicity (ADCC): After the antibody component of ADCs binds to the tumor cell antigen, the Fc portion can bind to Fc receptors on effector cells such as natural killer (NK) cells and macrophages. This triggers an ADCC response, leading to the direct killing of cancer cells (Trail and Bianchi, 1999). (3) Signal Inhibition and Apoptosis Induction: ADC antibodies can also bind to antigen targets on cancer cells, inhibiting downstream signaling pathways and inducing apoptosis (Senter, 2009). TV is an ADC that targets tissue factors, which are specifically expressed in CC tissues, making it a novel ADC target antigen. Additionally, the Vedotin component of TV includes a cytotoxic payload, namely, monomethyl auristatin E (MMAE), a microtubule inhibitor, enhancing its therapeutic potency (Francisco et al., 2003). It has been revealed that the MMAE in Vedotin can induce immunogenic cell death and modulate the tumor microenvironment by up-regulating PD-L1 expression in tumor cells, making them more susceptible to immunotherapy (Aschenbrenner, 2022; Heitz et al., 2023; Gray et al., 2023; Heiser et al., 2024). As a result, there is growing interest in the combination of TV with pembrolizumab. Despite the focus on TV monotherapy in this meta-analysis, emerging evidence suggests that combination strategies may further improve efficacy. In the innovaTV 205 trial (Vergote et al., 2023), the ORR and median duration of response of TV combined with pembrolizumab as second- or third-line therapy were 35.3% and 14.1 months, respectively; while those of TV monotherapy were 24% and 8.3 months in the innovaTV 204 study (Coleman et al., 2021). TV also showed additive effects when combined with carboplatin in the first-line setting (ORR 54.5%). These results indicate that combination regimens may offer enhanced benefits, particularly in immunotherapy-naïve patients or earlier treatment lines. The ongoing phase 3 innovaTV 301 trial further demonstrated that TV monotherapy provided a significant survival advantage over physician’s choice chemotherapy (OS 11.5 vs. 9.5 months), reinforcing its role as a backbone for future therapeutic combinations (Vergote et al., 2024). However, the potential for increased toxicity and the need for biomarker-driven patient selection highlight that the optimal application of TV, whether as monotherapy or in combination, remains to be defined (Vergote et al., 2023).

Our study reported a high incidence of AEs (99.1%) and AEs at Grades ≥3 (61.7%). This safety profile is comparable to other treatments in the second-/third-line setting, such as pembrolizumab (55.3%) and chemotherapy (62.3%) in the KEYNOTE-158 and innovaTV 301 trials (Vergote et al., 2024), respectively. Notably, TV-related toxicities (primarily ocular toxicities, bleeding, and neuropathy) are mechanistically distinct and generally manageable with prophylactic measures such as corticosteroid eye drops and cold compresses. In addition, proactive patient selection based on baseline ocular health, bleeding risk, and performance status may mitigate toxicity. The relatively low discontinuation rate due to AEs (14.8%) further supports the clinical feasibility of TV (Arn et al., 2023; Luu et al., 2023; Karpel et al., 2023; Agostinelli et al., 2023). Additionally, biomarker-driven patient selection is a crucial strategy to improve efficacy and reduce adverse effects. In ADC therapy, researchers focus on the identification of patients who will benefit most from TV treatment and specific biomarkers that can be employed to predict efficacy. This contributes to the more precise selection of appropriate patients in clinical practice, thus providing personalized treatment plans.

This may be the first scientific attempt to integrate clinical data on the use of TV in the treatment of r/mCC by a systematic review and meta-analysis, providing a comprehensive evaluation of its efficacy and safety and offering strong support for clinical decision-making. However, there are some limitations to this study. Firstly, the number of included studies is relatively small, which may limit the statistical power and generalizability of the findings. Secondly, all included studies are single-arm trials without control groups, which inherently increases the risk of bias and limits comparative interpretation. Although the MINORS tool is employed to assess methodological quality and random-effects models are used where appropriate, residual bias cannot be fully excluded. Thirdly, due to the lack of individual patient-level data, such important covariates as age, prior treatment history, or PD-L1 status are not adjusted through meta-regression. While subgroup analyses are conducted based on study-level characteristics (e.g., study design, sample size, and geographic location), no statistically significant interaction effects are observed, and heterogeneity remains low to moderate. Additionally, real-world data (RWD), which may provide insights into the effectiveness and safety of TV in broader patient populations, are not available during this study. Finally, potential publication bias cannot be fully ruled out, as unpublished data and conference abstracts are excluded due to limited reporting quality. These limitations highlight the need for future large-scale, real-world, or randomized comparative studies to validate and expand on our findings.

## 5 Conclusion

TV has demonstrated significant efficacy in the second-line and third-line treatment of r/mCC, including improvements in median OS, median PFS, and ORR. Although the incidence of AEs, particularly AEs at Grades 3–5, is relatively high during treatment, the overall safety profile is within acceptable limits. Nevertheless, it is still required to explore and validate its long-term efficacy and safety, as well as relevant strategies to optimize its application in clinical practice.

## Data Availability

The original contributions presented in this study are included in the article/Supplementary Material. Further inquiries can be directed to the corresponding authors.

## Supplementary material

SUPPLEMENTARY TABLE 1 Subgroup Analyses of Objective Response Rate (ORR), Disease Control Rate (DCR), the Incidence of Adverse Events (AEs) at Grades ≥3, and Progression-free Survival (PFS).
